# Observation on the therapeutic effect of laser acupuncture combined with Schroth therapy on adolescent idiopathic scoliosis

**DOI:** 10.12669/pjms.40.6.8636

**Published:** 2024-07

**Authors:** Zhaoqi Wang, Lei Lu, Liheng Wang

**Affiliations:** 1 Zhaoqi Wang, Department of Physiatry, The Second People’s Hospital of Dalian, Dalian 116011, Liaoning, China; 2 Lei Lu, Department of Physiatry, The Second People’s Hospital of Dalian, Dalian 116011, Liaoning, China; 3 Liheng Wang, Department of Physiatry, The Second People’s Hospital of Dalian, Dalian 116011, Liaoning, China

**Keywords:** Adolescent idiopathic scoliosis, Laser acupuncture, Scoliosis

## Abstract

**Objective:**

To explore the clinical effect of laser acupuncture combined with Schroth therapy on adolescent idiopathic scoliosis (AIS).

**Method:**

This was a retrospective study. Eighty AIS patients were admitted to The Second People’s Hospital of Dalian from March 2021 to March 2022 and divided into control group and experimental group according to the treatment method, with 40 cases in each group. The control group received Schroth therapy, and the experimental group received Schroth therapy and laser acupuncture therapy (MLS® laser). All treatments are performed five times a week for four consecutive weeks we compared the clinical effects of the two groups before treatment, six months and 12 months after treatment, and compared the improvement of Cobb angle, axial trunk rotation (ATR), musculoskeletal stiffness(The PulStarG3 system), and gait evaluation (Micro-Electro-Mechanical System(MEMS) between the two groups of patients.

**Result:**

After four weeks of treatment, the spinal condition of both groups of patients improved. After treatment, the experimental group showed greater improvement in Cobb angle, ATR, spinal range of motion, gait parameters, and clinical efficacy compared to the control group (p<0.05).

**Conclusion:**

Laser acupuncture combined with Schroth therapy is safe and effective in the treatment of AIS, and is more effective in correcting scoliosis and related problems of AIS.

## INTRODUCTION

Adolescent idiopathic scoliosis (AIS) is a rotational bending disease of the spine that occurs in a three-dimensional plane of unknown etiology and complexity. Scoliosis is defined by the American Scoliosis Research Society (SRS) as a Cobb’s method to measure the spinal curvature angle of standing spine X-ray films that is greater than 10°. AIS is most likely to occur in children aged 10-16 years, and the incidence in the adolescent population is about 2-2.5%.^1^ AIS mainly affects the spine and adjacent soft tissue structures, leading to various physical problems, such as abnormal posture, hunchback, decreased spinal flexibility, back pain, chest tightness, etc.^2^ As the Skeleton of adolescents is still growing, if the treatment is not timely, unprofessional or untreated, scoliosis will progress rapidly, which may further increase and compress the heart and lungs, leading to cardiopulmonary dysfunction. Severe scoliosis may even lead to high paraplegia, affecting daily life. At the same time, AIS can affect the physical appearance of adolescents, causing patients to feel inferior, anxious, and autistic. In severe cases, it can lead to mental illness and reduce the quality of daily life of adolescents.^3^

At present, AIS treatment is mainly aimed at controlling symptoms, delaying or preventing the progress of scoliosis. The main methods are divided into non-surgical treatment and surgical treatment. When the Cobb angle is greater than 40°, the international consensus is that surgical treatment is the first choice because of the poor effect of Conservative management. For patients with a Cobb angle less than 40°, non-surgical treatment is often performed, including exercise therapy, chiropractic therapy, or brace therapy.^4^ Exercise therapy is one of the commonly used treatment methods to reduce spinal and chest stiffness in AIS patients, including Schroth therapy, FITS therapy, Lyon therapy, Dobomed therapy, and SEAS therapy.^5^ Among them, Schroth therapy is the most extensively studied and widely used, based on the use of specific postures, sensory movements, and breathing exercises for scoliosis to improve patient movement control of posture. Research has confirmed that Schroth therapy can reduce Cobb angle, slow down the progression of spinal curve deterioration, and alleviate back pain.^6^

Acupoint stimulation has been proven to have clinical effects on restoring spinal morphology and alleviating musculoskeletal pain.^7^ Laser acupuncture is a new technology that uses low-intensity laser beam to irradiate and stimulate acupoints. Compared with traditional acupuncture, laser acupuncture has no direct contact with patients, which can avoid the breakage of needle body, stabbing organs or local infection in traditional acupuncture. It has the advantages of non-invasive, painless, safer and easier operation.^8^ Laser acupuncture has a wide range of clinical applications. It has achieved good clinical results in the treatment of various diseases, and is easier for beginners to operate.^9^ However, there is no clinical study on the application of laser acupuncture in AIS, especially no study comparing the clinical effect of Schroth therapy and laser acupuncture combined with Schroth therapy in AIS. The purpose of this study was to compare the difference between Schroth therapy and laser acupuncture combined with Schroth therapy on Cobb angle, axial trunk rotation (ATR), spinal mobility, gait parameters and clinical efficacy of AIS patients, so as to provide clinical reference for laser acupuncture treatment of AIS.

## METHODS

This was a retrospective study. Eighty patients with adolescent idiopathic scoliosis (AIS) admitted to The Second People’s Hospital of Dalian from March 2021 to March 2022, and the 80 patients were divided into control group(n=40) and experimental group (n= 40) according to different treatment methods.

### Ethical Approval

The study was approved by the Institutional Ethics Committee of Baoding No.1 Hospital (No.: 2020-10, date: January 10, 2020), and written informed consents were obtained from all participants and their guardians.

The control group received Schroth therapy, while the experimental group received Schroth therapy and laser acupuncture therapy. The design of individual Schroth therapy is based on the curve patterns classified by Schroth. The Schroth classification criteria include four curve patterns, which are determined based on the position of the curve, the importance of lumbar and thoracic vertebral protrusions, and the impact of scoliosis on the pelvis.^10^ The Scheros curve classification and standardized treatment plan is shown in Table-I. Laser acupuncture therapy uses dual wavelength, high-power infrared laser (Multiwave Locked System (MLS®) laser, Mphi, ASA srl, Vicenza, Italy), Laser acupuncture points include DU2, GB30, DU9, SP6, LR8, DU6, DU4, DU12. The laser punctured acupuncture points were treated with the handheld optical group according to the syndrome differentiation of traditional Chinese medicine. The specific treatment parameters were: 900Hz, FPW mode, 60 seconds per point, 29.12J, spot size 3 cm^2^, energy density 27J/cm^2^. All treatments are performed five times a week for four consecutive weeks.

**Table-I:** Baseline clinical and demographic characteristics of groups.

		Control	trial	*P* value
Age		13.23±2.76	13.71±3.01	0.18
Gender	Female	23	25	0.65
	Male	17	15	
Curve type	Right Thoracic	17	14	0.76
	left Thoracic	15	18	
	Right Thoracic-Left lumbar	8	8	

### Inclusion criteria:

- Adolescent idiopathic scoliosis patients without history of trauma.
- Positive Adam’s Forward Bend Test, Cobb Angle>10°.

### Exclusion criteria:

- Patients with incomplete general information or who withdrew from the study
- Patients with mental disorder or cognitive impairment
- Patients with open fractures
- Patients with severe dysfunction of heart, liver, kidney, lung and other visceral organs
- Patients with coronary heart disease, diabetes and other basic diseases
- Patients who were assessed as intolerable to surgery
- Patients who received conservative treatment and accompanied by obvious nerve damage.

### Measurements

Observe the Cobb angle, ATR, musculoskeletal stiffness, and gait evaluation of two groups of patients before treatment, six months and 12 months after treatment, and evaluate the clinical efficacy of the two regimens at six and 12 months after treatment. Cobb angle measurement with X-ray: select the vertebral body with the largest tilt at the head and tail side of the scoliosis segment as the end vertebra, make a straight line along the upper edge of the upper vertebra and the lower edge of the lower vertebra, and the included angle of the two perpendicular lines is the cobb angle. All Cobb angle measurements are made by an expert physician using the same Protractor three times to take the average of these measurements.

ATR measured by the same Physiotherapist with Adam’s Forward bending test, where the examiner sits behind the subject, with their line of sight parallel to the most prominent uneven height on the subject’s back. The patient is instructed to stand naked with their feet together, knees straight, arms naturally sagging, and slowly bend forward. The Scoliometer middle groove is placed on the spinous process, and the examiner places one finger directly above the groove. The data corresponding to the mercury particles are read to quantitatively evaluate the chest rotation angle and waist rotation angle. Using the PulstarG3 system (Sense Technology Inc) to evaluate the musculoskeletal stiffness at each vertebral level: a handheld pulse head is pressed against the patient and a single low energy pulse is provided to the vertebral level of interest.

The force sensor in the pulse head measures the resistance to the pulse and transmits the analysis results to a digital computer, The digital computer can be considered as the computer assisted spinal palpation in a series of Bar chart (the red Bar chart shows abnormal musculoskeletal hardness, and the green Bar chart shows normal musculoskeletal hardness). The evaluation criteria for efficacy are: cure, disappearance of scoliosis after treatment, Cobb angle<5°, normal physiological curvature, and normal function; Effective, symptoms and physiological curvature improved, Cobb angle decreased ≥5°, ATR decreased≥2°; Invalid, symptoms and scoliosis did not improve significantly before and after treatment, and even worsened, with a decrease in Cobb angle of<5°. Effective rate of treatment= (cured+effective)/total number of cases × 100%.^11^

Gait data is collected using wearable devices ((Micro-Electro-Mechanical System, MEMS) and transmitted to a computer server according to the manufacturer’s protocol. The sampling frequency is set to 20Hz20 Hz. The collected gait parameters include average pressure distribution, stride length, stride time, and walking speed.

### Statistical analysis

SPSS 22.0 was used for statistical analysis, with measurement data expressed as *χ̅*±*S*. Two independent sample t-tests were conducted, and counting data was expressed as n(%). Inter group comparisons were performed using χ². The test showed a statistically significant difference of P<0.05.

## RESULTS

According to the inclusion criteria, a total of 80 AIS patients were included in the study, including a control group of 40 patients, 23 females, and 17 males; There are 40 patients in experimental group, 25 females and 15 males. The baseline clinical and demographics characteristics of the two groups of patients are shown in Table-I. There was no significant difference in age, gender, and scoliosis type between the two groups of patients (p>0.05).

Before treatment, there was no significant difference in the Cobb angle of scoliosis, ATR, and musculoskeletal stiffness between the two groups of patients (p>0.05). After 6 and 12 months of treatment, the degree of scoliosis in both groups of patients improved, with the experimental group showing more improvement in Cobb angle and ATR compared to the control group. The red bars of musculoskeletal stiffness in the experimental group were also lower; In addition, the involvement of spinal structure in the experimental group improved significantly, and the improvement of scoliosis in the experimental group at six months of treatment reached the level of control group at 12 months of treatment. The above differences were statistically significant (all P<0.05), (Table-II).

**Table-II:** Scores for the Cobb angle, angle of trunk rotation, musculoskeletal stiffness between Baseline and post-treatment interventions.

	Baseline				Post-treatment (6 month)				Post-treatment (12 month)			

	Control	trial	t	P	control	trial	t	P	control	trial	t	P
Cobb-T(°)	17.98±3.67	18.43±4.67	0.15	0.88	15.13±2.34	13.98±2.31	2.21	0.03*	13.21±3.21#	8.99±2.19##	6.87	0.00*
Cobb-L(°)	15.89±3.53	16.03±3.35	0.02	0.98	13.22±2.11	12.34±2.91	1.55	0.13	12.98±3.02	9.12±2.32##	6.41	0.00*
ATR-T(°)	9.34±2.23	9.93±3.02	0.31	0.76	7.12±2.38	6.23±2.19	1.74	0.08*	6.34±3.12	4.57±1.89##	3.07	0.00
ATR-L(°)	4.53±1.93	5.02±2.12	0.34	0.74	3.78±2.09	3.45±1.12	0.88	0.38	3.01±1.78	2.12±0.79	2.89	0.01*
Red bars per patient	15.67±2.65	16.45±2.19	1.44	0.16	11.98±2.11	8.53±1.98	7.54	0.00*	8.34±2.18##	2.78±0.71###	15.34	0.00*

***Note:*** Compared with the control group,

* P<0.05, and compared with the same group after treatment (6 months), #P<0.05.

Before treatment, both groups of patients had a longer left foot stride and stride time in right thoracic scoliosis, while the right foot in the left thoracic vertebrae had a longer stride time. After six months of treatment, the stride length and stride time of both groups improved, and the stride length and stride time of the experimental group were shorter than those of the corresponding control group, while the walking speed was higher than that of the control group (all P<0.05). After 12 months of treatment, the stride length, stride time, and walking speed of the two groups continued to improve, but the difference was not significant (p>0.05), (Table-III).

**Table-III:** Characteristic of gait patterns in experimental group and control group.

	Baseline				Post-treatment (6 month)				Post-treatment (12 month)			

	control		Trial		control		Trial		control		Trial	

	Left	Right	Left	Right	Left	Right	Left	Right	Left	Right	Left	Right
***Stride length, m***												
Right Thoracic	0.79±0.09	0.65±0.11	0.75±0.04	0.61±0.09	0.83±0.10	0.73±0.09*	0.90±0.09*	0.80±0.11*	0.94±0.18**#	0.90±0.21**#	1.24±0.17**#	1.11±0.15*#
left Thoracic	0.67±0.13	0.74±0.06	0.68±0.08	0.75±0.04	0.75±0.09*	0.83±0.10*	0.84±0.06*	0.92±0.09**	0.95±0.12**#	1.01±0.14**#	1.19±0.23**#	1.22±0.27*#
Right Thoracic-Left lumbar	0.74±0.05	0.76±0.06	0.73±0.07	0.75±0.10	0.80±0.19	0.82±0.21	0.85±0.13*	0.86±0.16**	1.01±0.19**#	1.01±0.14**#	1.21±0.22**#	1.27±0.28*#
***Stride time, seconds***												
Right Thoracic	1.98±0.11	1.78±0.13	1.93±0.12	1.73±0.19	1.78±0.21**	1.65±0.18*	1.65±0.23	1.54±0.19*	1.38±0.12*##	1.29±0.16*##	1.23±0.18*#	1.20±0.16*#
left Thoracic	1.74±0.12	1.95±0.14	1.75±0.12	1.96±0.11	1.58±0.21**	1.64±0.19**	1.67±0.13*	1.77±0.18*	1.24±0.12*##	1.35±0.17*#	1.35±0.18*#	1.40±0.19*#
Right Thoracic-Left lumbar	1.76±0.13	1.79±0.11	1.78±0.17	1.76±0.19	1.68±0.18*	1.71±0.28	1.50±0.21*	1.57±0.17*	1.29±0.21*##	1.18±0.21*#	1.28±0.23*#	1.19±0.21*#
***Walking velocity, m/s***												
Right Thoracic	0.75±0.11		0.70±0.21		0.85±0.15**		0.94±0.21***		1.23±0.19**#		1.31±0.25**##	
left Thoracic	0.71±0.18		0.74±0.16		0.83±0.16**		0.91±0.18**		1.28±0.18**#		1.36±0.23**##	
Right Thoracic-Left lumbar	0.72±0.21		0.69±0.27		0.79±0.19**		0.82±0.22**		1.12±0.22**#		1.15±0.25**##	

***Notes:*** Compared with baseline in the same group,

* P<0.05, Compared with Post-treatment (6 month) in the same group, #P<0.05.

After six months of treatment, the total effective rates of the two groups were 32.5% and 50%, respectively, with no statistically significant difference(p>0.05). After 12 months of treatment, the total effective rates of the two groups were 77.5% and 95%, respectively, with a statistically significant difference (p<0.05), and it was better than those of six months after treatment (P<0.05), (Table-IV).

**Table-IV:** Trial clinical efficacy between groups.

Group	Cure	Improvement	Ineffective	total efficiency	P value
Post-treatment (6 month)					
Control	0 (0%)	13 (32.5)	27 (67.5%)	13 (32.5%)	0.17
Trial	0 (0%)	20 (50%)	20 (50%)	20 (50%)	
Post-treatment (12 month)					
Control	5 (12.5%)	26 (65%)	9 (22.5%)	31 (77.5%)	0.04
Trial	11 (27.5%)	27 (67.5%)	2 (5%)	38 (95%)	

***Notes:*** Compared with Post-treatment (6 month) in the same group, *P<0.05.

## DISCUSSION

This study showed that the Cobb angle of both groups of patients decreased after treatment, which is consistent with other research results.^12^ Research has confirmed that Schroth therapy is effective in stabilizing the degree of mild scoliosis (Cobb angle less than 15.3° and axial torso rotation angle less than 8.9°).^13^ At the same time, the results of this study also showed that the ATR angle of AIS patients was significantly improved after treatment, especially in the experimental group, the improvement degree of scoliosis at the time of six months of treatment had reached the level of 12 months of treatment in the control group, indicating that the clinical effect of laser acupuncture combined with Schroth therapy on AIS was significantly better than that of Schroth therapy alone. Because acupuncture treatment can alleviate pain, alleviate inflammatory reactions, and improve the balance of strength around the spine by unblocking meridians, regulating the circulation of qi and blood. This study confirms that Schroth therapy has a positive effect on the treatment of AIS, reducing Cobb angle and ATR angle, improving gait patterns, which is consistent with the results of multiple clinical studies.^14^

Research has shown that acupuncture and massage, including traditional Chinese medicine, can play an important role in the treatment of AIS.^15^ The mechanism of acupuncture treatment for AIS is that acupuncture can promote the patient’s body’s qi and blood circulation, better transport and absorption of nutrients, improve local metabolism and circulation, and promote the recovery of diseased tissues. Peng reported that the treatment effect mainly based on acupuncture treatment is superior to that of simple treatment.^3^ Others have reported that electroacupuncture treatment can relax paravertebral muscles and alleviate pain.^16^

The mechanism of massage therapy for AIS is to adjust muscle tension through manipulation, alleviate muscle tension on both sides of the spine, strengthen nerve center transmission and trunk muscle strengthening, induce correct coordinated movement and posture control mode, promote muscle balance on both sides of the spine, and improve scoliosis. Although acupuncture and massage cannot cure AIS, they can regulate the balance of spinal biomechanics, alleviate the tension of muscles around the spine, and have a certain corrective effect. They can serve as effective auxiliary treatment measures for AIS. Schroth therapy mainly involves opening the concave side of the scoliosis through different body postures to bring the spine back to the midline, which has a positive impact on the degree of scoliosis and the improvement of patient quality of life.^17^,^18^

Laser acupuncture is a new technology that uses laser irradiation to stimulate acupoints. It has the functions of analgesia, enhancing anti-inflammatory, promoting tissue repair, expanding blood vessels and enhancing body immunity, compared with traditional acupuncture, it has the advantages of less trauma, safety and painlessness.^19^ Although laser acupuncture is widely used in clinical practice at this stage, there is no systematic evaluation report on the efficacy and safety of laser acupuncture in the treatment of AIS patients. The purpose of this study is to evaluate the efficacy and safety of laser acupuncture in the treatment of AIS patients. Cobb angle and ATR angle are important predictive factors for good prognosis in AIS patients.^20^

The results of this study showed that the total efficacy of the experimental group was higher than that of the control group at six and 12 months after treatment. It shows that the combination of laser acupuncture therapy and Schroth therapy can quickly correct scoliosis, significantly enhance the clinical effect, and make AIS patients recover quickly.

### Limitations of this study

However, this study also has some shortcomings, such as small sample size, small course of treatment, and short follow-up time. It still needs further clinical research to observe the long-term clinical effect of laser acupuncture therapy combined with Schroth therapy on AIS, so as to apply a better scheme to patients in need.

## CONCLUSION

Laser acupuncture therapy combined with Schroth therapy have clinical effects on the treatment of AIS, which can effectively prevent the curve progression of AIS patients. The combination of the two therapies can significantly enhance the clinical effect, shorten the recovery time of patients, and the clinical application is safe and reliable, which is worthy of clinical promotion.

### Authors’ Contributions:

**ZW and LL:** Carried out the studies, data collection, drafted the manuscript, are responsible and accountable for the accuracy and integrity of the work.

**LW:** Performed the statistical analysis and participated in its design; All authors read and approved the final manuscript.
